# Optical Coherence Tomographic Features and Prognostic Values of Macular Edema in Vogt-Koyanagi-Harada Disease

**DOI:** 10.3389/fmed.2021.772439

**Published:** 2022-01-10

**Authors:** Peng Qin, Zi Ye, Guannan Su, Aize Kijlstra, Peizeng Yang

**Affiliations:** ^1^The First Affiliated Hospital of Chongqing Medical University, Chongqing Key Lab of Ophthalmology, Chongqing Eye Institute, Chongqing Branch of National Clinical Research Center for Ocular Diseases, Chongqing, China; ^2^University Eye Clinic, Maastricht University, Maastricht, Netherlands

**Keywords:** macular edema, Vogt-Koyanagi-Harada disease, OCT feature, prognostic value, inner-segment/outer-segment junction

## Abstract

**Purpose:** To determine optical coherence tomographic (OCT) features of macular edema (ME) and identify potential prognostic values for ME and visual outcomes in Vogt-Koyanagi-Harada disease (VKH).

**Methods:** In the retrospective case series, a total of 1,377 VKH patients who were seen in a tertiary uveitis center between September 2011 and January 2018 were reviewed on their demographics, visual acuity, ocular and extraocular manifestations, modes of treatment, and OCT examinations. Of these patients, 79 (5.7%) having ME were included for analysis of OCT features. Four patients were missed without ME resolution, and the remaining 75 patients who either had ME resolved or were followed up for 2 years were included for analysis of disease outcomes.

**Results:** Of the 115 affected eyes in these 79 patients, 100 (87.0%) had cystoid ME (CME), accounting for the most common OCT feature of VKH-related ME. Disruption of the inner-segment/outer-segment junction (IS/OS) band seen in 33 (28.7%) affected eyes of 24 (30.4%) patients was found as a risk factor for the development of persistent ME [10 of 62 (16.1%) vs. 13 of 13 (100%); *P* < 0.001] and a poor visual outcome (1.16 ± 0.42 vs. 1.17 ± 0.46 in logMAR unit; *P* = 0.89). CME patients with a concurrent choroidal neovascular membrane often had a disrupted IS/OS band, thus becoming refractory cases. A 6-month well-controlled intraocular inflammation following standard treatment regimens was found to associate with complete resolution of the refractory edema [4 of 5 (80%) vs. 2 of 13 (15%); *P* = 0.02].

**Conclusions:** Intraretinal cystoid changes are most commonly seen in the edematous macula of VKH patients. Disruption of the IS/OS band is a useful risk sign for poor ME and visual outcomes in VKH-related ME, and a long-term well-controlled intraocular inflammation may be critical for the resolution of refractory cases.

## Introduction

Vogt-Koyanagi-Harada (VKH) disease is characterized by bilateral granulomatous uveitis associated with auditory, neurological, and integumentary impairments. It is thought to be a T-cell mediated autoimmune disease (1–3) that targets antigens associated with melanocytes. It is more prevalent in Asia, Latin America, and the Middle East (3). VKH often presents acutely with diffuse choroiditis, serous retinal detachment and disk edema, and eventually develops into a granulomatous anterior uveitis associated with a sunset glow fundus in the chronic phase. Patients treated timely with adequate doses of corticosteroids and immunomodulatory agents, usually achieve a rapid resolution of both ocular and systemic inflammation, often resulting in a good visual outcome (4, 5). However, chronic disease may develop if no adequate treatment is instituted (6), leading to complications such as cataract, glaucoma, macular edema (ME), choroidal neovascular membrane (CNV), and chorioretinal atrophy, which may cause a poor visual outcome over the years.

ME, an accumulation of fluid in the macula, is often associated with intraocular inflammation, resulting in significant visual impairments (7). The incidence, time course, and visual outcome of uveitic ME may vary and depend on the underlying etiology, site, and nature of the inflammation. It is more commonly seen in posterior or intermediate uveitis, particularly in retinal vasculitis, but is not frequently reported in patients with VKH disease (8, 9). A study on 111 Japanese VKH patients showed that ME was observed in 1.4% of the affected eyes (10). Our previous study on 1,468 Chinese VKH patients showed that 3.8% of the affected eyes had ME (11). An understanding of optical coherence tomographic (OCT) features of ME in VKH patients may provide information on the underlying etiology as well as the visual outcome. However, the relatively low incidence of ME in eyes of VKH patients and lack of long-term follow-up limits our understanding of OCT features and prognostic values of this sight-threatening condition, and was therefore the purpose of this study.

In the present study, we retrospectively reviewed 79 VKH patients who had been diagnosed with ME in our uveitis center. Features of ME as detected by OCT as well as prognostic values for both ME and visual outcomes were evaluated.

## Materials and Methods

We retrospectively reviewed the case records of all VKH patients seen in the uveitis center of the First Affiliated Hospital of Chongqing Medical University from September 2011 to January 2018. A total of 1,377 patients were diagnosed with VKH disease according to the VKH International Committee criteria revised in 2001 as well as the criteria proposed by us previously (12, 13). An eye was defined as having ME if the central retinal thickness (CRT) was more than 260 μm as detected by OCT. Eighty-three VKH patients who had bilaterally visible fundi were diagnosed with ME, as based on OCT findings. Of these patients, 79 were included in this study and the remaining patients were excluded due to concurrent diseases that might result in macular abnormalities, including hypertension, diabetes, or high myopia. The study adhered to the tenets of the Declaration of Helsinki and was approved by the Ethics Committee of the First Affiliated Hospital of Chongqing Medical University. Informed consent for using clinical data retrospectively has been waived by the Ethics Committee given the fact that all the data extraction were performed without patient identifiers.

Medical records of the patients were reviewed, and data were collected on patient demographics, best corrected visual acuity (BCVA), the onset and clinical course of disease, recurrent episodes of intraocular inflammation, ocular and extraocular manifestations, modes of therapy, and OCT examinations.

OCT examinations were performed with a Spectralis OCT (Heidelberg Engineering, Heidelberg, Germany) by experienced operators at the initial visit to our uveitis center and during subsequent follow-up visits if abnormalities of the posterior segment were suspected. Measurement of the CRT and identification of the fovea were carried out by the automated protocol of the OCT machine. Fluorescein angiography and OCT angiography were performed when a CNV was suspected according to OCT images. The following OCT features were recorded: (1) diffuse retinal thickening; (2) cystoid macular edema (CME); (3) posterior hyaloidal traction; (4) epiretinal membrane; (5) integrity of outer retinal layers [particular inner-segment/outer-segment junction (IS/OS)]; and (6) concurrent CNV and subretinal fluid.

Treatments included adequate doses of systemic corticosteroids combined with steroid-sparing agents as described previously elsewhere (14). Doses were gradually tapered over a period of more than 1 year to reduce relapse rate as described earlier (14). Briefly, VKH patients received either early regimen (ER) with a higher initial dose (0.6–0.8 mg/kg/day) of prednisone if they were seen within the first 2 months after uveitis onset, or late regimen (LR) with a lower initial dose (0.4–0.6 mg/kg/day) if they were referred to our clinic 2 months after uveitis onset. Cyclosporine was the most commonly used steroid-sparing agent, and the initial dose was 2–4 mg/kg/day. Other drugs used in combination with steroids included cyclophosphamide, chlorambucil and methotrexate. The initial dose of prednisone was usually used for 1–2 weeks in all patients and then gradually tapered to 15–20 mg/day over 4–6 months. Systemic corticosteroids were normally used at this dose for 4–6 months, then tapered to 10 mg/day over 2–3 months and finally to a stop over another 2–3 months. Cyclosporine, if the initial dose was more than 2 mg/kg/day, was also gradually tapered to a maintenance dose (2 mg/kg/day) over 5–6 months, and was maintained at this dose in combination with systemic corticosteroids for another 6–12 months. Corticosteroid eye drops, mydriatic and cycloplegic drops were applied in patients with recurrent granulomatous anterior uveitis. Severe ME was treated with one or two triamcinolone acetonide injections, either subtenonally or intravitreally. Anti-vascular endothelial growth factor (VEGF) agent (conbercept ophthalmic injection, 0.05 mL, Chengdu Kanghong Biotechnologies Co. Ltd, China) was administered intravitreally one to three times in eyes with a concurrent CNV.

Statistical analysis was performed with Prism 8.3 (GraphPad Software, San Diego, CA, USA). For all analyses in this study, the OCTs of only 1 eye were evaluated so as to minimize the risk of correlation between eyes in individual patients who had bilateral simultaneous ME. Here, we arbitrarily chose the right eye. For univariate analysis, the Mann-Whitney test was performed for numerical data, while the χ2 test was employed for categorical data. For analysis of the BCVA and CRT values at various timepoints, we used the paired *t*-test. For comparison between groups, one-way ANOVA followed by the Bonferroni-Dunn test was used. For bivariate correlation analysis, the Spearman Rho test was employed to evaluate the nonparametric data. A 2-tailed *P*-value of < 0.05 was considered as statistically significant.

## Results

Of the 79 patients included in the study, 42 (53.2%) were male. The mean age at diagnosis with VKH disease was 40.6 years (range 19–71 years). The mean duration of VKH disease at the onset of ME was 9.8 months (range 5–16 months). Systemic findings, including integumentary, auditory and neurologic impairments, were found in 35 (44.3%), 40 (50.6%), and 44 (55.7%) of these patients, respectively. There were 27 (34.2%) patients with a BCVA of 20/200 or worse in their affected eyes at the onset of ME. Twenty-nine (36.7%) patients were given a higher dose of prednisone in the ER group, whereas the other 50 (63.3%) patients received a lower dose of prednisone in the LR group. All the patients displayed concurrent anterior segment inflammation as evidenced by aqueous flare and cells, and mutton fat keratic precipitates when diagnosed with ME, suggestive of at least one recurrent episode of intraocular inflammation in these cases. Twenty-three patients who had severe macular edema were treated with one or two triamcinolone acetonide injections.

**Figure 1:**
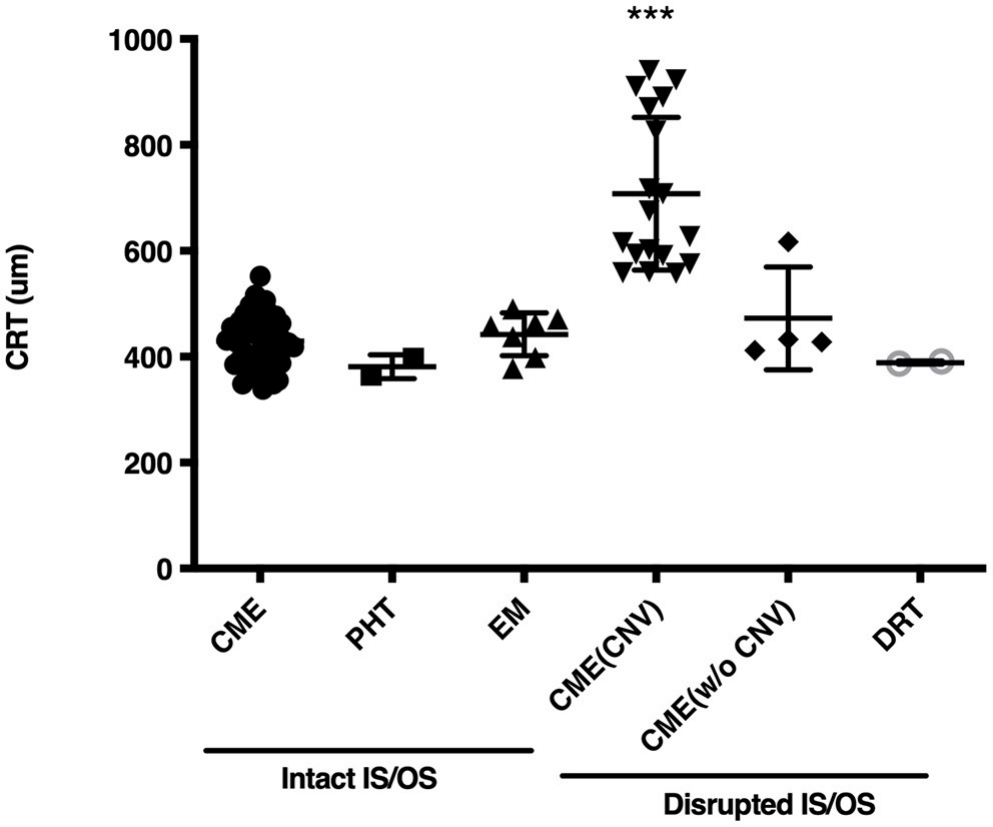
Central retinal thickness (CRT) in eyes of Vogt-Koyanagi-Harada disease patients at the onset of macular edema. CME, cystoid macular edema; PHT, posterior hyaloidal traction; EM, epiretinal membrane; CNV, choroidal neovascular membrane; CME (CNV), CME with a CNV; CME (w/o CNV), CME without a CNV; DRT, diffuse retinal thickening; IS/OS, inner-segment/outer-segment junction. ***indicates *P* < 0.001 as compared to every other group.

The OCT images of these 79 patients at their ME onset were reviewed, and showed that 115 eyes had clinically significant ME. Of the 115 affected eyes, 100 (87.0%) had CME, and 10 (8.7%) had an epiretinal membrane, and the rest 5 (4.3%) either had posterior hyaloidal traction or diffuse retinal thickening (Table 1). Compromised IS/OS integrity was often observed, whereby 65 (82.3%) patients with 82 (71.3%) affected eyes had an intact IS/OS band, while 24 (30.4%) patients with 33 (28.7%) affected eyes had a disrupted IS/OS band. There were 10 patients who had bilateral ME with distinct OCT features and were thus counted twice. Among the patients with a disrupted IS/OS band, 18 (22.8%) with 26 (22.6%) affected eyes had a concurrent CNV, and 11 (13.9%) with 15 (13.0%) affected eyes also showed the presence of subretinal fluid. At the onset of ME, the CRT was higher in CME cases with a concurrent CNV (707.8 ± 144.0 μm), than in cases with other OCT patterns (430.7 ± 53.4 μm; *P* < 0.001) (Figure 1).

**Table 1:** Optical coherence tomographic features of ME in eyes with VKH disease.

**Optical coherence**	**Patients**	**Number of affected**
**tomographic features**	**(%)**	**eyes (%)**
Total	79^a^	115
Intact IS/OS	65 (82.3)	82 (71.3)
CME	55 (69.6)	69 (60.0)
Posterior hyaloidal traction	3 (3.8)	3 (2.6)
Epiretinal membrane	7 (8.9)	10 (8.7)
Disrupted IS/OS	24 (30.4)	33 (28.7)
CME with a CNV	18 (22.8)	26 (22.6)
CME without a CNV	4 (5.1)	5 (4.3)
Diffuse retinal thickening	2 (2.5)	2 (1.7)

a *There were 10 patients who had simultaneous bilateral ME with distinct optical coherence tomographic features*.

*ME, macular edema; VKH, Vogt-Koyanagi-Harada; IS/OS, inner-segment/outer-segment junction; CME, cystoid macular edema; CNV, choroidal neovascular membrane*.

To examine risk factors for developing persistent ME in VKH patients, we defined the persistent ME as those cases where it was either not or partially resolved at a 2-year follow-up timepoint. Four patients were missed when their ME was not resolved, and the remaining 75 patients who either had ME resolved or were followed up for at least 2 years were included in the following analyses. In 62 patients the ME had completely resolved, and 13 patients still showed persistent ME at the 2-year timepoint. Neither demographics nor systemic manifestations were found to be risk factors for the development of persistent ME (Table 2). Neither a BCVA of 20/200 or worse at ME onset [19 (30.6%) vs. 7 (53.8%); *P* = 0.11] nor treatments of the LR group [38 (61.3%) vs. 8 (61.5%); *P* = 0.99] were shown to associate with the outcome of persistent ME. Among the OCT features, a disrupted IS/OS band [10 (16.1%) vs. 13 (100%); *P* < 0.001], particularly, the CME with a concurrent CNV [5 (8.1%) vs. 13 (100%); *P* < 0.001], was a significant risk factor for the development of persistent ME. Since none of the subjects with an intact IS/OS band had persistent ME, we were unable to perform a multivariate logistic model that included and estimated the relative contributory effects of IS/OS disruption on persistent ME development.

**Table 2:** Demographic and optical coherence tomographic risk factors for developing persistent ME in VKH patients.

**Risk factors**	**Completely resolved ME**	**Not or partially resolved ME**	***P***
	**(62 patients)**	**(13 patients)**	
Mean age of VKH onset in years (range)	42.1 (23–71)	37.9 (19–68)	0.91^a^
Mean duration of VKH before ME onset in months (range)	9.7 (6–15)	10.9 (5–16)	0.89^a^
Male sex, no. (%)	34 (54.8)	7 (53.8)	0.95^b^
Integumentary findings, no. (%)	27 (43.5)	6 (46.2)	0.86^b^
Auditory impairments, no. (%)	32 (51.6)	5 (38.5)	0.39^b^
Neurologic findings, no. (%)	36 (58.1)	7 (53.8)	0.78^b^
Optical coherence tomographic features^c^			
Disrupted IS/OS, no. (%)	10 (16.1)	13 (100)	<0.001^b^
CME, no. (%)	51 (82.3)	13 (100)	0.10^b^
Concurrent CNV, no. (%)	5 (8.1)	13 (100)	<0.001^b^
Posterior hyaloidal traction, no. (%)	2 (3.2)	0 (0)	0.51^b^
Epiretinal membrane, no. (%)	7 (11.3)	0 (0)	0.20^b^
Diffuse retinal thickening, no. (%)	2 (3.2)	0 (0)	0.51^b^
BCVA of 20/200 or worse at ME onset^c^, no. (%)	19 (30.6)	7 (53.8)	0.11^b^
Treatments of the late regimen group, no. (%)	38 (61.3)	8 (61.5)	0.99^b^

a *Mann-Whitney test*.

b *χ2 test*.

c *For patients with simultaneous bilateral ME, only the right eyes were included for analysis*.

*ME, macular edema; VKH, Vogt-Koyanagi-Harada; IS/OS, inner-segment/outer-segment junction; CME, cystoid macular edema; CNV, choroidal neovascular membrane; BCVA, best-corrected visual acuity*.

The effects of IS/OS integrity on duration of ME in patients with VKH disease were also investigated (Supplementary Table 1). CME in patients with an intact IS/OS band was often resolved within 3 months [31 (41.3%) vs. 12 (16.0%); *P* < 0.001]. CME in cases who had a concurrent CNV often required a longer time to resolve and tended to be persistent [5 (6.7%) vs. 13 (17.3%); *P* = 0.008], thus becoming refractory cases. Once the ME had completely resolved, none of our patients had a relapse of ME during their follow-ups.

To evaluate the effects of IS/OS integrity on the outcomes of BCVA and CRT, we further examined the BCVA and CRT at the timepoints of both ME onset and ME resolution or final visit if persistent ME existed (Table 3). For the patients with an intact IS/OS band, the CRT was found to decrease at the timepoint of ME resolution when compared to that at ME onset (217.8 ± 21.5 vs. 436.1 ± 52.4 μm; *P* < 0.001) and was accompanied with an improved BCVA [in logarithm of the minimum angle of resolution (logMAR) units, 0.35 ± 0.23 vs. 0.85 ± 0.41; *P* < 0.001]. For the patients with a disrupted IS/OS band, some had their ME resolved and these cases showed a significantly decreased CRT (228.7 ± 15.7 vs. 563.8 ± 68.6 μm; *P* < 0.001) and an improvement in their BCVA (0.86 ± 0.20 vs. 1.08 ± 0.38; *P* = 0.04) at the timepoint of ME resolution. Moreover, the persistent ME occurred in some patients with a disrupted IS/OS band, although a significantly decreased CRT (440.1 ± 91.6 vs. 740.9 ± 145.3 μm; *P* < 0.001) was observed at the time of their last visit. No significant changes in BCVA were found in these patients (1.16 ± 0.42 vs. 1.17 ± 0.46; *P* = 0.89).

**Table 3:** Mean CRT and BCVA (in logMAR units) at various timepoints.

**Features^**a**^**	**BCVA (logMAR)^b^**				**CRT (μm)^b^**			
	**ME onset**	**ME resolution**	**Final visit**	***P*** **^c^**	**ME onset**	**ME resolution**	**Final visit**	***P*** **^c^**
Intact IS/OS	0.85 ± 0.41	0.35 ± 0.23	–	<0.001	436.1, 52.4	217.8 ± 21.5	–	<0.001
Disrupted IS/OS							
Resolved ME	1.08 ± 0.38	0.86 ± 0.20	–	0.05	563.8, 68.6	228.7 ± 15.7	–	<0.001
Persistent ME	1.17 ± 0.46	–	1.16 ± 0.42	0.89	740.9, 145.3	–	440.1 ± 91.6	<0.001

a *For patients with simultaneous bilateral macular edema (ME), only the right eyes were included for analysis*.

b *Data were expressed as mean ± standard deviation*.

c *Comparison of parameters between the timepoints of ME onset and ME resolution/final visit*. *CRT, central retinal thickness; BCVA, best-corrected visual acuity; logMAR, logarithm of the minimum angle of resolution; ME, macular edema; IS/OS, inner-segment/outer-segment junction*.

As the patients with a disrupted IS/OS band experienced a longer duration of ME and were at high risk of progression to persistent ME and poor visual outcomes, we focused on these patients and found that 13 out of the 18 patients with CME and a concurrent CNV ultimately developed persistent ME. At the onset of ME, the height of the CNV did not show a significant correlation with the thickness of the ME (*R* = 0.4605; *P* = 0.06) (Supplementary Figure 1), suggesting that the CNV lesion area was not proportional to the severity of ME in these cases. The etiology and severity of this refractory ME might be caused by a combination of various factors. Recurrent intraocular inflammations may play a role in persistent ME development. Analysis of these 18 patients showed a significant difference (*P* < 0.01) in the longest duration of the convalescent phase between the five patients with resolved ME (7.4 ± 1.5 months) and the remaining 13 patients with persistent ME (4.5 ± 0.7 months), suggesting that the 13 patients might experience more recurrent episodes of intraocular inflammation. A mean duration of the convalescent phase before resolution of ME in these 5 patients was 6.4 ± 0.9 months, suggesting that a well-controlled intraocular inflammation for at least 6 months may be a cut-off period for blood-retinal barrier restoration and resolution of refractory ME. Univariate analysis showed that an achievement of a 6-month well-controlled intraocular inflammation following the standard treatment regimen was a prognostic factor for the resolution of refractory ME [4 of 5 (80%) vs. 2 of 13 (15%); *P* = 0.02) (Table 4). According to our observations, the refractory ME resolved in some cases with a long-term well-controlled intraocular inflammation, despite the presence of a CNV (Supplementary Figure 2). Interestingly, the CNV remained quiescent by the end of their follow-ups. Besides the standard treatment regimen used for the intraocular inflammation, five of the 18 patients also received 1–3 intravitreal anti-VEGF injections, which induced CNV regression in all these patients. However, only two of these five patients had their ME resolved, suggesting the CNV regression following anti-VEGF therapy may not be a prognostic factor for the resolution of refractory ME [2 of 5 (40%) vs. 3 of 13 (23%); *P* = 0.58]. Detailed information of the patients receiving anti-VEGF therapy is shown in Supplementary Table 2.

**Table 4:** Prognostic factors for the resolution of ME with a concurrent CNV in VKH patients.

**Prognostic factors^**a**^**	**Completely resolved ME (5 patients)**	**Not or partially resolved ME (13 patients)**	***P***
A 6-month well-controlled intraocular inflammation, no. (%)	4 (80.0)	2 (15.4)	0.02^b^
The CNV regression, no. (%)	2 (40.0)	3 (23.1)	0.58^b^

a *For patients with simultaneous bilateral ME, only the right eyes were included for analysis*.

b *χ2 test*.

*ME, macular edema; CNV, choroidal neovascular membrane; VKH, Vogt-Koyanagi-Harada*.

## Discussion

In the present study we examined the OCT data of ME in VKH and identified intraretinal cystoid changes as the most common OCT feature in this disease. The presence of IS/OS junction abnormalities as shown by spectral OCT was strongly associated with the development of persistent ME as well as a poor visual outcome. An achievement of a 6-month well-controlled intraocular inflammation was found to be of prognostic value for the resolution of refractory ME in VKH.

ME is an important cause of visual loss in patients with uveitis and OCT provides a real-time *in-vivo* cross-sectional image of the edematous macula and is widely used in the diagnosis and management of ME (15, 16). Since ME is only encountered in ~2–6% of VKH (11), only limited research has been published on OCT features of ME in this disease. Our retrospective study on OCT features of ME in 115 affected eyes of 79 VKH cases is, to our knowledge, the largest published cohort so far. The CME, which was observed in the majority (87.0%) of the affected eyes, was the most common OCT feature of ME in VKH. As the integrity of the outer retinal hyper-reflective bands as seen on OCT was shown to correlate with vision (17), we further examined the integrity of the IS/OS band which reflects the structure of photoreceptors in the retina. To our understanding, as VKH is a choroidal disease, and due to the close proximity to the choroid, the outer retinal layers were supposed to be more susceptible to inflammatory insults. However, only around one third of the patients had a disrupted IS/OS band as shown on OCT, and most of these cases had a concurrent CNV. The underlying pathogenesis of ME secondary to VKH disease remains largely unknown. The OCT findings were in accordance with our clinical observations (11, 14) that most VKH patients may have a good visual prognosis after control of intraocular inflammation and resolution of ME, unless a CNV has formed and the damages to photoreceptors have ensued (11). Subretinal fibrosis is an important complication of VKH and might invade the macula. There were 3 VKH patients who had macular edema and subretinal fibrosis in this study. However, subretinal fibrosis was found to reside outside the macula and did not show any leakage in fluorescein angiography (data were not shown).

Disruption of the IS/OS band, particularly in CME cases with a concurrent CNV, was shown to be a risk factor for the development of persistent ME. During inflammation, choroidal vessels were reported to become dilated and prone to leaking fluid (18), which was also seen for the CNVs. Moreover, disruption of the IS/OS band in eyes of VKH patients implicated a compromise of the photoreceptors, resulting in a worse visual outcome in these patients. In this case, visual loss might be irreversible, even after resolution of the edema. Our results showed that more than half of our patients with a BCVA of 20/200 or worse at the onset of ME were those with a disrupted IS/OS band. Additionally, unlike patients with an intact IS/OS band who had a significantly decreased CRT and an improved BCVA at ME resolution, the patients with a disrupted IS/OS band showed no improvement in BCVA, despite a significantly reduced CRT at the resolution of ME, suggesting that disruption of the IS/OS band may also be an important risk factor for a poor visual outcome. Previous study (11) found treatments of the LR group as a risk factor for developing CME. According to our observation in this study, most VKH patients with CME had their edema resolved within 3 months, and treatments of the late regimen group were not found to associate with the incidence of persistent macular edema.

The VKH patients with persistent ME showed shorter duration of the convalescent phase and experienced an increased number of recurrent episodes of intraocular inflammation, suggesting that the persistent ME may be attributable to (1) persistent low-grade expression of intraocular inflammatory cytokines; (2) vulnerable blood-retinal barrier damaged by chronic inflammation. On a univariate analysis, an achievement of a 6-month well-controlled intraocular inflammation, rather than the CNV regression following anti-VEGF therapy, was found as a prognostic factor for the resolution of refractory ME in VKH patients, suggesting that a long-term control of intraocular inflammation would be of help in restoration and maintenance of the blood-retinal barrier. Moreover, several inflammatory cytokines can trigger the VEGF pathway (7, 19–21), thus emphasizing the critical importance of a strict-controlled intraocular inflammation for any patients undergoing anti-VEGF therapy for the treatment of refractory uveitic ME. In this regard, a sustained local release of anti-inflammatory agents might induce appreciable benefits. Recently, a dexamethasone implant and a fluocinolone acetonide implant have been approved for the treatment of non-infectious posterior uveitis (22, 23). It would be interesting to investigate whether combination therapies of such steroid implants with anti-VEGF agents might lead to an improved treatment of ME in VKH eyes.

Limitations include a small sample size and the retrospective nature. Due to the limited number of VKH patients who had ME, we reviewed the patients back to Sep 2011. However, new treatment regimens have emerged over the past 10 years, which may also affect the outcome of persistent ME. Moreover, as anti-VEGF agents for treating both uveitic ME and CNV were off-label use in China, only a small number of VKH patients received 1 to 3 anti-VEGF injections for the treatment of ME associated with a CNV. We lack data to show whether the patients could gain better outcomes from intensive anti-VEGF therapies.

In conclusion, CME was the most common OCT feature of VKH-related ME. VKH Patients with disruption of the IS/OS band in the edematous macula were at high risk for the development of persistent ME and were subject to longer duration of edema and a poor visual outcome. A long-term well-controlled intraocular inflammation following the standard treatment regimen may benefit the resolution of refractory ME in VKH.

## Data Availability Statement

The raw data supporting the conclusions of this article will be made available by the authors, without undue reservation.

## Ethics Statement

The studies involving human participants were reviewed and approved by the Ethics Committee of the First Affiliated Hospital of Chongqing Medical University. Written informed consent for participation was not required for this study in accordance with the national legislation and the institutional requirements.

## Author Contributions

PQ: acquisition and interpretation of data, statistics, and writing of manuscript. ZY and GS: acquisition of data. AK: writing of manuscript. PY: design of the study, supervision, and writing of manuscript. All authors contributed to the article and approved the submitted version.

## Funding

The work was supported by Chongqing Outstanding Scientists Project (2019), Chongqing Chief Medical Scientist Project (2018), Chongqing Key Laboratory of Ophthalmology (CSTC, 2008CA5003), and Chongqing Science & Technology Platform and Base Construction Program (cstc2014pt-sy10002). The sponsor or funding organization had no role in the design or conduct of this research.

## Conflict of Interest

The authors declare that the research was conducted in the absence of any commercial or financial relationships that could be construed as a potential conflict of interest.

## Publisher's Note

All claims expressed in this article are solely those of the authors and do not necessarily represent those of their affiliated organizations, or those of the publisher, the editors and the reviewers. Any product that may be evaluated in this article, or claim that may be made by its manufacturer, is not guaranteed or endorsed by the publisher.

## Abbreviations

BCVA: best corrected visual acuity
CME: cystoid macular edema
CNV: choroidal neovascular membrane
CRT: central retinal thickness
IS/OS: inner-segment/outer-segment junction
logMAR: logarithm of the minimum angle of resolution
ME: macular edema
OCT: optical coherence tomography
VEGF: vascular endothelial growth factor
VKH: Vogt-Koyanagi-Harada.
