# Supplementary material for: Optical Coherence Tomographic Features and Prognostic Values of Macular Edema in Vogt-Koyanagi-Harada Disease
Source: Front Med (Lausanne). 2022 Jan 10;8:772439. doi: 10.3389/fmed.2021.772439 (PMC8785902; doi:10.3389/fmed.2021.772439)
Supplement: Supplementary file 1 [file Data_Sheet_1.docx]

**Supplementary Material**

**Supplementary Table 1.** Duration of ME in eyes with VKH Disease.

**Supplementary Table 2.** Information of the patients receiving intravitreal conbercept therapy.

**Supplementary Figure 1.** Correlation between the height of elevated CNV and the onset thickness of edematous macula in eyes with Vogt-Koyanagi-Harada disease

**Supplementary Figure 2.** Optical coherence tomography macular scans from selected patients

| **Supplementary Table 1. Duration of ME in eyes with VKH Disease.** | | | | | | |
| --- | --- | --- | --- | --- | --- | --- |
| **Optical coherence tomographic features^a^** | | **=< 3 months** | **>3 months;**  **=<12 months** | **>12 months;**  **=<24 months** | **>24 months** | **P** |
| **Total, no. (%)** | | **32(42.7)** | **21(28.0)** | **9(12.0)** | **13(17.3)** |  |
| **Intact IS/OS** | |  |  |  |  |  |
|  | CME, no. (%) | 31(41.3) | 12(16.0) |  |  | <0.001^b^ |
|  | Posterior hyaloidal traction, no. (%) |  | 2(2.7) |  |  | - |
|  | Epiretinal membrane, no. (%) | 1(1.3) | 6(8.0) |  |  | 0.007^b^ |
| **Disrupted IS/OS** | |  |  |  |  |  |
|  | CME (with a CNV), no. (%) |  |  | 5(6.7) | 13(17.3) | 0.008^b^ |
|  | CME (without a CNV), no. (%) |  |  | 3(4.0) |  | - |
|  | Diffuse retinal thickening, no. (%) |  | 1(1.3) | 1(1.3) |  | - |
| ^a^For patients with simultaneous bilateral ME, only the right eyes were included for analysis. | | | | | | |
| ^b^χ2 test.  Abbreviations: ME, macular edema; VKH, Vogt-Koyanagi-Harada; IS/OS, inner-segment/outer-segment junction; CME, cystoid macular edema; CNV, choroidal neovascular membrane. | | | | | | |

| **Supplementary Table 2. Information of the patients receiving intravitreal conbercept therapy.** | | | | | | | | |
| --- | --- | --- | --- | --- | --- | --- | --- | --- |
| **Patient number/**  **age(years)/**  **gender** | **Duration of ME at initial injection of Conbercept**  **(months)** | **Number of Conbercept injections** | **Location of CNV membrane** | **Size of CNV(DD)** | **Regression of CNV** | **Resolution of ME** | **Frequency of recurrent inflammation during 2-year follow-ups** | **Treatments at initial time of injection** |
| 1/47/F | 3 | 1 | Juxtafoveal | 1.5 | Yes | Yes | 5 | CsA, prednisone, topical corticosteroids, STTA |
| 2/32/M | 2 | 3 | Subfoveal | 1 | Yes | No | 8 | MTX, prednisone, topical corticosteroids, STTA |
| 3/25/F | 1 | 1 | Juxtafoveal | 0.5 | Yes | No | 7 | Prednisone, topical corticosteroids, STTA |
| 4/51/F | 4 | 3 | Subfoveal | 1 | Yes | Yes | 6 | MTX, prednisone, topical corticosteroids, STTA |
| 5/40/M | 0.5 | 2 | Juxtafoveal | 2 | Yes | No | 8 | CsA, prednisone, topical corticosteroids, IVTA |
| Abbreviations: ME, macular edema; F, female; M, male; CNV, choroidal neovascular membrane; DD, disc diameter; CsA, Cyclosporine; MTX, methotrexate; STTA, sub-tenon triamcinolone acetonide; IVTA, intravitreal triamcinolone acetonide. | | | | | | | | |


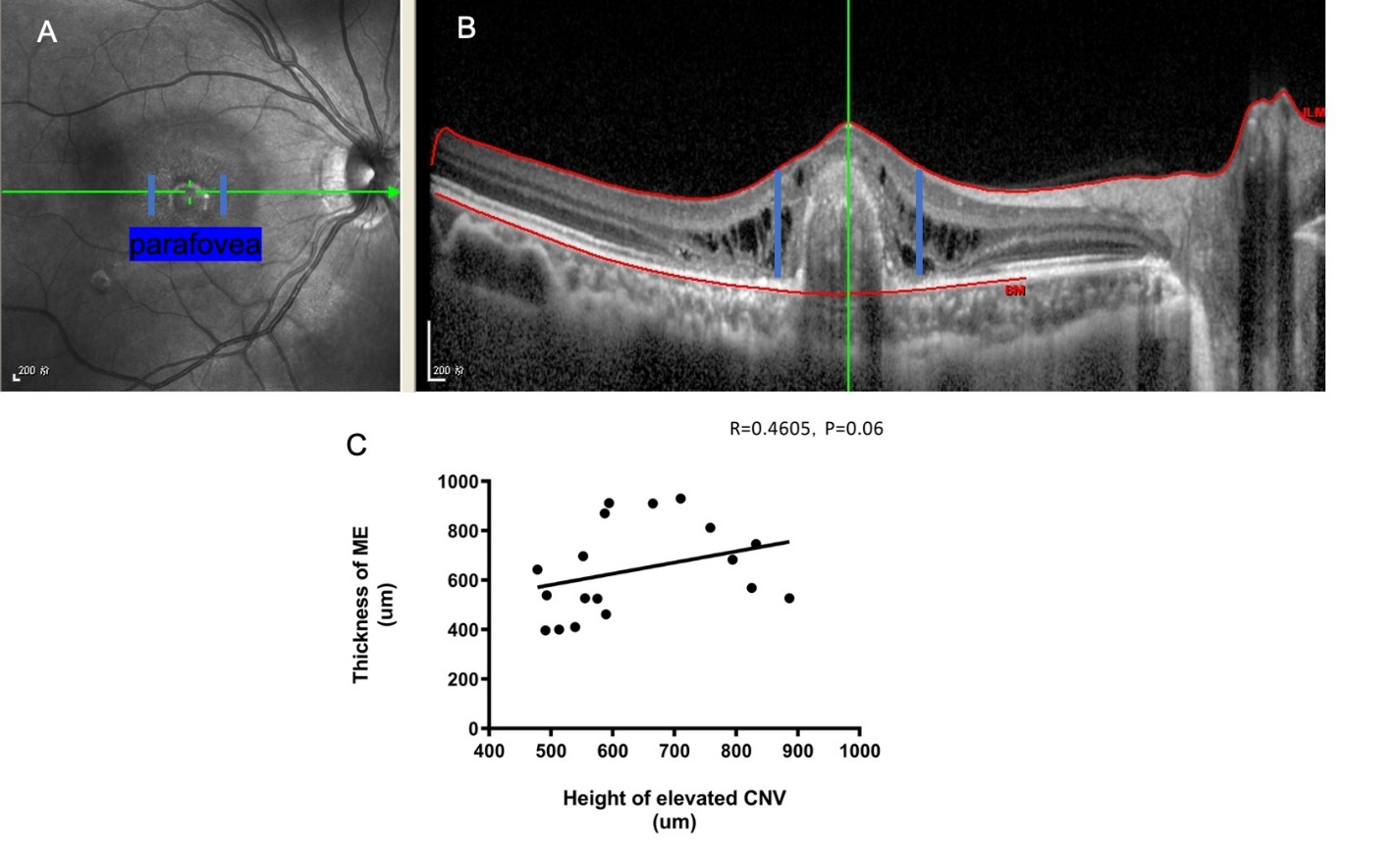


**Supplementary Figure 1. Correlation between the height of elevated CNV and the onset thickness of edematous macula in eyes with Vogt-Koyanagi-Harada disease.**

The blue lines in A indicate the parafoveal area that corresponds to the measurement sites of the edematous macula in the retinal section shown in B. The green arrow in A indicates the optical section that has been taken and shown in B. The green line in B shows the elevated CNV. ME, macular edema; CNV, choroidal neovascular membrane.


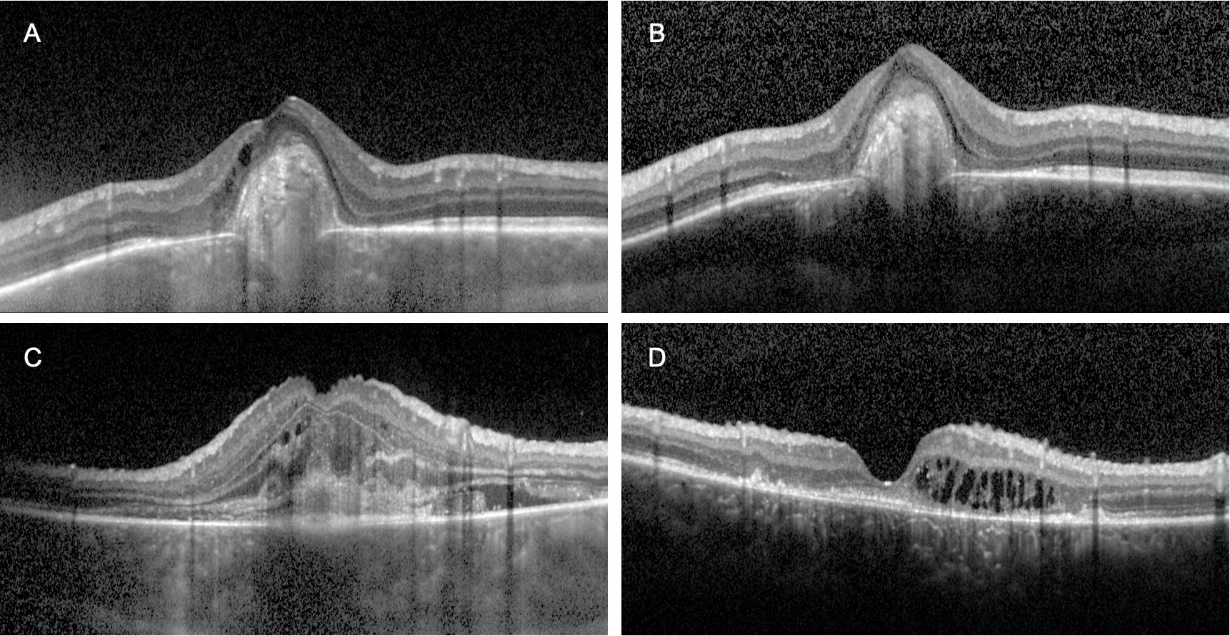


**Supplementary Figure 2. Optical coherence tomography macular scans from selected patients.**

Optical coherence tomography macular scans from selected patients showing ME and a concurrent CNV, at ME onset (A, C) and by the end of follow-up (B, D). Macular scans of the patient with a 6-month well-controlled intraocular inflammation are shown in A and B, while scans of the patient who had regression of CNV following intravitreal injections of conbercept are shown in C and D. ME, macular edema; CNV, choroidal neovascular membrane.
